# The influence of infant feeding attitudes on breastfeeding duration: evidence from a cohort study in rural Western Australia

**DOI:** 10.1186/s13006-015-0048-3

**Published:** 2015-08-21

**Authors:** Kylee N. Cox, Roslyn C. Giglia, Colin W. Binns

**Affiliations:** School of Public Health, Curtin University, Bentley, Western Australia 6102 Australia; UWA Centre for Child Health Research, University of Western Australia, Crawley, Western Australia 6009 Australia

## Abstract

**Background:**

Breast milk is the optimal source of nutrition for infants in the first six months of life. Promoting and protecting breastfeeding is reflected in public health policy across the globe, but breastfeeding rates in both developing and industrialised countries continue to demonstrate that few mothers meet these recommendations. In addition to sociodemographic factors such as age, education and income, modifiable factors such as maternal infant feeding attitudes have been shown to influence breastfeeding duration. The objective of this paper was to describe the influence of infant feeding attitudes on breastfeeding duration in rural Western Australia.

**Methods:**

A cohort of 427 women and their infants were recruited from hospitals in rural Western Australia and followed for a period of 12 months. Information about feeding methods was gathered in hospital and at a further seven follow-up contacts. Infant feeding attitude was measured using the Iowa Infant Feeding Attitude Scale (IIFAS), and a score of > 65 was considered positive towards breastfeeding.

**Results:**

Mothers with an IIFAS score of > 65 were approximately twice as likely to be exclusively breastfeeding at six months, and breastfeeding at any intensity to 12 months. The median duration of exclusive breastfeeding for mothers with an IIFAS score of > 65 was 16 weeks (95 % CI 13.5, 18.5) compared with 5 weeks for those with a score < 65 (95 % CI 3.2, 6.8) (*p* < 0.0001). The median duration of any breastfeeding to 12 months was more than twice as long for mothers with an IIFAS score > 65 (48 vs. 22 weeks, *p* < 0.001).

**Conclusions:**

Women in this rural cohort who had a more positive attitude towards breastfeeding had a longer duration of both exclusive breastfeeding to six months and any breastfeeding to 12 months. Further research examining the breastfeeding attitudes of specific subgroups such as men, grandparents and adolescents in rural areas will contribute to the evidence base and help to ensure that breastfeeding is seen as the normal method of infant feeding.

## Background

Breast milk is the optimal source of nutrition for infants in the first six months of life [1] and the promotion and protection of breastfeeding is reflected in public health policy across the globe. Significant gains in breastfeeding duration have been made, with 50 % of infants in the least developed countries aged 0 - 5 months exclusively breastfed in 2012, compared to 38 % in 2000 [2]. In developed countries, considerable variation in breastfeeding duration exists, however the evidence suggests few mothers meet the World Health Organization’s recommendations [3–5]. In Australia, despite near universal breastfeeding initiation rates, it is estimated that approximately 17 % of infants are exclusively breastfed to six months of age [6].

Influences on infant feeding in developed countries are varied, but include sociodemographic factors such as maternal age, level of education and family income [3, 7–9]. Whilst these variables are strong predictors of breastfeeding practice, they are not amenable to modification and are therefore difficult to address through breastfeeding promotion interventions [10]. It has been suggested that a focus on modifiable factors such as maternal infant feeding attitude may help to increase breastfeeding duration [11–15].

### Breastfeeding attitudes

A mother’s attitude towards breastfeeding as the choice of infant feeding method has been shown to influence the likelihood of her initiating and continuing to breastfeed [11, 15–17]. This attitude is shaped by factors such as personal breastfeeding experience support from social networks including her close friends and family, particularly her mother and partner [10, 18–20]. Additionally, exposure to positive role models and societal values which promote breastfeeding as normal and desirable contribute to positive breastfeeding attitude [12, 21].

The infant feeding attitudes of significant social supports such as a woman’s partner and mother have also been cited as positive influences on breastfeeding practice [11, 22–26]. Women have traditionally learned about infant feeding from their mothers, through positive role modelling and practical problem solving [27]. Similarly, a woman’s partner contributes both practical and emotional support for breastfeeding and studies have found that greater engagement of fathers in the antenatal period increases the likelihood that breastfeeding is initiated and maintained [24, 28]. In rural communities where women may be geographically isolated from their own mothers or other childbearing peers, the support of fathers as breastfeeding advocates may be more influential.

In addition to personal experience and beliefs, breastfeeding attitudes may be shaped by cultural or personal experiences, although there is limited evidence regarding differences in attitudes between urban and rural areas of developed countries. In a study of factors associated with breastfeeding initiation and duration in rural USA, the lack of role models and a perception that breastfeeding was not the normal infant feeding method for women of particular cultural backgrounds were identified as potentially contributing factors [29]. Other evidence suggests that economic factors in rural communities such as the need for women to return to work and limited community facilities to support continued breastfeeding are also significant barriers [30].

Measurement of maternal infant feeding attitude has been the subject of extensive research and validated tools have been developed to quantify the effect on breastfeeding practice. The use of the Iowa Infant Feeding Attitudes Scale (IIFAS) [16] is one tool that has been reliably replicated in a number of settings to determine the strength of infant feeding attitude [11, 12, 18, 29, 31]. Research has demonstrated that not only does the IIFAS predict maternal infant feeding attitude [12, 13, 15, 18], but is also an accurate measure of paternal attitudes [11, 24, 32].

Whilst there is a significant body of literature that documents the measurement of maternal infant feeding attitude using the IIFAS in developed countries, there is little evidence of its use in rural populations. Psychological factors such as optimism and faith in breast milk were measured using a range of validated instruments in a prospective cohort study of 375 women giving birth in a large regional centre in rural Queensland [33]. The authors reported that women with greater faith in breast milk were 36 % more likely to be fully breastfeeding at six months (OR 1.36, 95 % CI 1.14, 1.63) and 70 % more likely to continue any breastfeeding to six months (OR 1.70, 95 % CI 1.26, 2.29) [33]. In an ethnographic study as part of the Family Life Project in rural North Carolina and Pennsylvania, mothers who did not initiate breastfeeding were less positive about breastfeeding and cited factors such as pain, embarrassment, lack of social acceptability and a planned return to work as reasons for not breastfeeding their infants [29].

The Rural Infant Feeding Study (RIFS) aimed to describe the factors associated with breastfeeding practice from birth to 12 months of age in rural Western Australia. The objective of this paper is to determine if infant feeding attitudes are independently associated with breastfeeding duration to 12 months for rural areas.

## Methods

### Sample selection

The Rural Infant Feeding Study (RIFS) was conducted in regional Western Australia between April 2010 and November 2012. The study aimed to determine information about infant feeding practices from women who lived in non-metropolitan communities (including regional centres, smaller towns and remote areas of the state). Rurality was defined based on the Australian Standard Geographical Classification (ASGC) Remoteness Structure [34] and all locations other than major cities were included. A sample of 489 mothers and their infants were recruited between April 2010 and November 2011 from hospitals with maternity service capacity in four non-metropolitan health regions of Western Australia (WA) and followed for 12 months from birth. Women who had delivered an infant without serious illness, who read and understood English and who resided in a regional area of WA were eligible to take part in the study. Mothers were contacted and invited to participate in the study by research staff with assistance from midwives at each hospital. Based on the prevalence of ‘any breastfeeding’ at six months determined in the Perth Infant Feeding Study Mark II (PIFS II) [7], an initial sample of 500 was estimated. Assuming power of 0.80 with 95 % level of confidence and allowing for approximately 20 % loss to follow-up, a final sample size of 400 for the main study was calculated using the StatCalc program of Epi Info™ [35] to detect a 50 % prevalence of ‘any breastfeeding’ at six months.

Whilst findings from the RIFS related to factors associated with breastfeeding at discharge from hospital and breastfeeding duration have been reported previously [36, 37], this paper provides a more detailed analysis of maternal feeding attitude as an independent influence on breastfeeding behaviour in the rural cohort under study.

### Data collection

The data collection tools were based on those used in the PIFS II [7] with additional questions included to ascertain geographical location and access to sources of information about infant feeding. The modified tools were pilot tested with a small group of rural mothers (*n* = 4) to assess suitability prior to use in the regional setting. Geographical location was used to ascribe a “remoteness score” using the Accessibility/Remoteness Index of Australia (ARIA) classification [38]. The score is based on road distance to regional service centres, using service centre population size as a proxy for availability of services. Breastfeeding terms and definitions in this study were consistent with those recommended by the World Health Organization [39] and adopted for use in Australia. An infant was considered to be exclusively breastfed if they had received only breast milk since birth, with the exception of drops or syrups consisting of vitamins, mineral supplements or medications. If an infant had received food or fluids other than breast milk at any point after birth, they were no longer considered exclusively breastfed. Infants who had received breast milk in addition to other fluids such as water, juice or oral rehydration fluids at any point after birth were considered to be fully (or predominantly) breastfed. Infants who received any breast milk (either exclusively or in addition to other food or fluids) were included in the calculation of ‘any breastfeeding’.

Once consent to participate was received, mothers were asked to complete a baseline questionnaire to determine demographic characteristics and feeding practices whilst in hospital. For mothers with internet access (87 %), a login and password were provided to enable the completion of the baseline and follow-up questionnaires online. Mothers without internet access (13 %) were contacted via telephone to complete the follow-up interviews. Over the 12 month period, mothers were contacted eight times (at birth, then at 4, 10, 16, 26, 32, 40 and 52 weeks) to determine changes to feeding methods, as well as factors that influenced their feeding decisions, including access to websites specifically designed to support breastfeeding. Mothers were asked at each time point if there had been any change to feeding method, including introduction of other fluids or food, since the previous survey. If mothers indicated that there had been a change, they were asked to provide the infants age in weeks when the change occurred. Mothers were contacted by email or phone within one week of the date that the follow-up survey was due. A further two reminder emails or phone calls were made in the following two weeks before a letter was sent to encourage contact with the researcher. If no response to this letter was received, the mother was considered lost to follow-up.

Maternal infant feeding attitude was measured in the baseline questionnaire using the Iowa Infant Feeding Attitudes Scale (IIFAS) [16], a 17 item scale which measures attitudes towards both breast and formula feeding with regards to the health and nutritional benefits, and the cost and convenience of each method. The IIFAS has been shown previously to be a valid and reliable measure of infant feeding attitudes among women in the USA [29], Scotland [12, 31] and Japan [15]. Each item is measured on a five point scale and items more favourable to formula feeding are reverse-scored to give an overall score. Potential scores range from 17 (indicating attitudes that favour formula feeding) to 85 (indicating attitudes that favour breastfeeding). The scale has been used extensively in cohort studies to determine maternal attitudes to breastfeeding [7, 11, 18, 40] although there is limited evidence of its use in rural settings.

The perceived infant feeding preferences of the baby’s father and maternal grandmother were also measured in the baseline questionnaire. To assess paternal feeding preference, mothers were asked to indicate if they believed their partner preferred breastfeeding, formula feeding, did not have a preference, or if they had not discussed it. Similarly, mothers were asked to indicate their own mothers feeding preference using the same responses.

### Data analysis

Data were analysed using the Statistical Package for Social Sciences (SPSS) Version 22 [41]. Univariate summary statistics were produced to describe the characteristics of the study sample. Chi-square tests were used to determine associations between variables of interest (maternal age, parity, maternal education, household income, maternal country of birth, birth weight and method of delivery).

Cox’s proportional hazards model was used to determine the effect of breastfeeding knowledge and attitude on duration of breastfeeding. Univariate logistic regression analysis was used to screen for potentially significant factors influencing breastfeeding duration. Association between breastfeeding practice (‘any’ and ‘exclusive’ breastfeeding) and the significant factors reported in univariate logistic regression analysis were then examined using Cox’s proportional hazards model, controlling for those significant predictors of breastfeeding practice determined in the literature and other potential demographic confounders. Backward elimination procedure was applied to obtain the final model in the regression analysis. The final model includes all variables included in the initial model, although the results presented are restricted to variables related to feeding attitude. All tests were two tailed and a p-value of less than 0.05 was considered statistically significant. Survival analysis was conducted to determine median breastfeeding duration using the Kaplan Meier technique in order to account for censored data, including participants who continued to breastfeed beyond the study period or who discontinued in the study. As survival analysis utilises censored data, which may be skewed, median duration gives a more accurate measure of central tendency. The internal consistency of the IIFAS was measured using Cronbach’s α, with an α of 0.7 or greater considered acceptable [42].

### Ethics

Ethics approval for this study was granted by the Curtin University Human Research Ethics Committee (SPH-0005-2008), the WA Country Health Service Ethics Committee (2009:25) and the St John of God Healthcare Ethics Committee (392).

## Results

### Participant characteristics

Of the 1170 women eligible to participate in the study, 819 were contacted and 489 women gave consent to participate. Of those women, 427 completed the baseline survey, representing 52 % of women contacted. Women who had been discharged from hospital in the first 24 h, who were not on the ward at the times that the researcher visited, or who had been identified as not appropriate to contact by midwifery or medical staff (for example if they had developed post-delivery complications) accounted for those not contacted.

The demographic characteristics of the participants are given in Table 1. The majority of mothers (82.6 %) were Australian born, and 94.5 % indicated that they were partnered (married or de facto). More than half of the participants (56.3 %) had an annual family income of AUD$72,800 or more, and 59.9 % indicated that they had private health insurance. Around one third of women (31.2 %) were not in the paid workforce in the six months prior to the study and 28.8 % indicated that they were on paid parental leave.

**Table 1:** Selected demographic characteristics of Rural Infant Feeding Study (RIFS) participants

Demographic characteristic (*n* = 427) (Mean ± SD)	*n*	%
Mean maternal age (yrs): 30.2 ± 5.2		
Mean birth weight (g): 3523 ± 471		
Mean gestational age (wks): 39.5 ± 1.4		
Health service region^a^ (*n* = 427)		
Midwest	291	68.1
South West	100	23.4
Wheatbelt	19	4.4
Goldfields	15	3.5
Pilbara	2	0.5
Remoteness classification (ARIA) (*n* = 424)		
HA (Highly Accessible)	19	4.4
A (Accessible)	259	60.7
MA (Moderately Accessible)	95	22.2
R (Remote)	31	7.3
VR (Very Remote)	20	4.7
Maternal age (yrs) (*n* = 427)		
<20	11	2.6
20-29	184	43.1
≥30	232	54.3
Parity (*n* = 424)		
Primiparous	178	41.7
Multiparous	246	57.6
Maternal education (*n* = 417)		
Did not complete high school	57	13.3
Completed Yr 12/technical/trade qualification	198	46.4
Bachelor degree or higher	162	37.9
Mother’s country of birth (*n* = 420)		
Australia/New Zealand	363	85.0
UK/Ireland	26	6.1
Other	31	7.3
Household income (AUD) (*n* = 409)		
<72,800	167	39.1
≥72,800	242	56.7
Birth weight (g) (*n* = 422)		
<2500	7	1.6
≥2500	415	97.2
Method of delivery (*n* = 425)		
Vaginal	244	57.1
Assisted (forceps/suction)	60	14.1
Caesarean section	121	28.3

^a^Western Australia Department of Health region

Compared with births in Western Australia in the corresponding time period, mothers in this study had a similar mean age 30.2 years (± standard deviation [SD] 5.2) vs. 29.6 years (±5.7, *p* = 0.02), and the mean birth weight of their infants was greater 3523g (±471) vs. 3337g (±601, *p* < 0.001). Fewer infants were delivered via caesarean section (28.3 % vs. 33.6 %, *p* = 0.026) and a greater proportion of mothers in the study were born in Australia or New Zealand (85.0 % vs. 72.0 %, *p* < 0.001). Compared with births in the regions under study, mothers in the study were older (*p* < 0.001), and more likely to be married (*p* = 0.02). There were also fewer women born in countries other than Australia or New Zealand (*p* < 0.001).

A total of 178 (41.6 %) mothers had incomplete data sets, having failed to complete all of the surveys at the specified time points after completion of the baseline questionnaire. Compared with the women who remained in the study, women lost to follow-up were more likely to be younger (*p* = 0.005), single (*p* = 0.005) and only have public health insurance (Medicare) (*p* = 0.005). They were also less likely to have a higher education degree (*p* = 0.006) and be employed in higher-level occupations (*p* = 0.037).

### Breastfeeding attitudes

Maternal breastfeeding attitudes were measured using the IIFAS scale as described above. The mean IIFAS score for all mothers participating in the study was 66 (range 39-84 ± SD 8.3). Further grouping of the responses to each of the 17 items in the IIFAS as positive (agreement), negative (disagreement) or neutral responses is presented in Table 2. The Cronbach’s α score of 0.75 indicates acceptable internal reliability of the scale in this sample [41].

**Table 2:** Rural Infant Feeding Study (RIFS) participants’ infant feeding attitudes using the Iowa Infant Feeding Attitudes Scale (IIFAS) (*n* = 427)

Item	Disagree^a^		Neutral		Agree^b^	
	(%)	*n*	(%)	*n*	(%)	*n*
1. The nutritional benefits of breast milk last only until the baby is weaned from breast milk	71.6	300	17.7	74	10.7	45
2. Formula-feeding is more convenient than breast-feeding	72.4	305	17.3	73	10.2	43
3. Breast-feeding increases mother-infant bonding	5.8	25	32.3	35	85.9	367
4. Breast milk is lacking in iron	62.3	266	30.9	132	5.2	22
5. Formula-fed babies are more likely to be overfed than breast-fed babies	26.9	115	41.7	178	31.4	134
6. Formula-feeding is the better choice if the mother works outside the home	50.8	217	33.5	143	15.7	67
7. Mothers who formula-feed miss one of the great joys of motherhood	33.2	142	24.6	105	42.2	180
8. Women should not breast-feed in public places such as restaurants	89.9	384	6.6	28	3.5	15
9. Babies who are fed breast milk are healthier than babies who are fed formula	25.3	108	31.6	135	43.1	184
10. Breast-fed babies are more likely to be overfed than formula-fed babies	64.9	277	31.8	136	3.3	14
11. Fathers feel left out if a mother breast-feeds	67.0	286	22.7	97	10.3	44
12. Breast milk is the ideal food for babies	4.4	19	8.0	34	87.6	374
13. Breast milk is more easily digested than formula	4.9	21	22.2	95	72.8	311
14. Formula is as healthy for an infant as breast milk	44.5	190	40.0	171	15.7	66
15. Breast-feeding is more convenient than formula-feeding	9.8	42	17.8	76	72.4	309
16. Breast milk is less expensive than formula	3.0	13	2.8	12	94.1	402
17. A mother who occasionally drinks alcohol should not breast-feed her baby	46.1	197	29.7	127	24.1	103

Note: The items 1, 2, 4, 6, 8, 10, 11, 14, and 17 were reversed when calculating the score

^a^ Disagree includes ‘strongly disagree’ and ‘disagree’

^b^ Agree includes ‘strongly agree’ and ‘agree’

The majority of mothers (94.1 %) agreed that breast milk was less expensive than formula, and 72.4 % agreed that breastfeeding was more convenient than formula feeding. A third of mothers (33.3 %) disagreed that formula feeding meant missing one of the great joys of motherhood, and a further 24.6 % had neutral feelings about this. Almost two thirds (62.1 %) disagreed that breastfeeding increased mother-infant bonding, and one quarter (25.3 %) disagreed that babies who were breastfed were healthier than formula-fed babies. More than half of mothers either agreed that infant formula was as healthy as breast milk (15.7 %) or expressed neutral feelings about the difference (40.0 %).

**Fig. 1:**
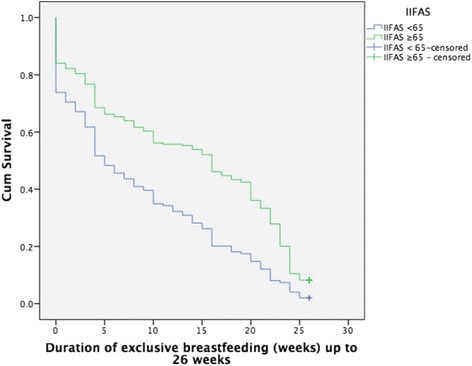
Exclusive breastfeeding duration to six months (26 weeks) by IIFAS score

**Fig. 2:**
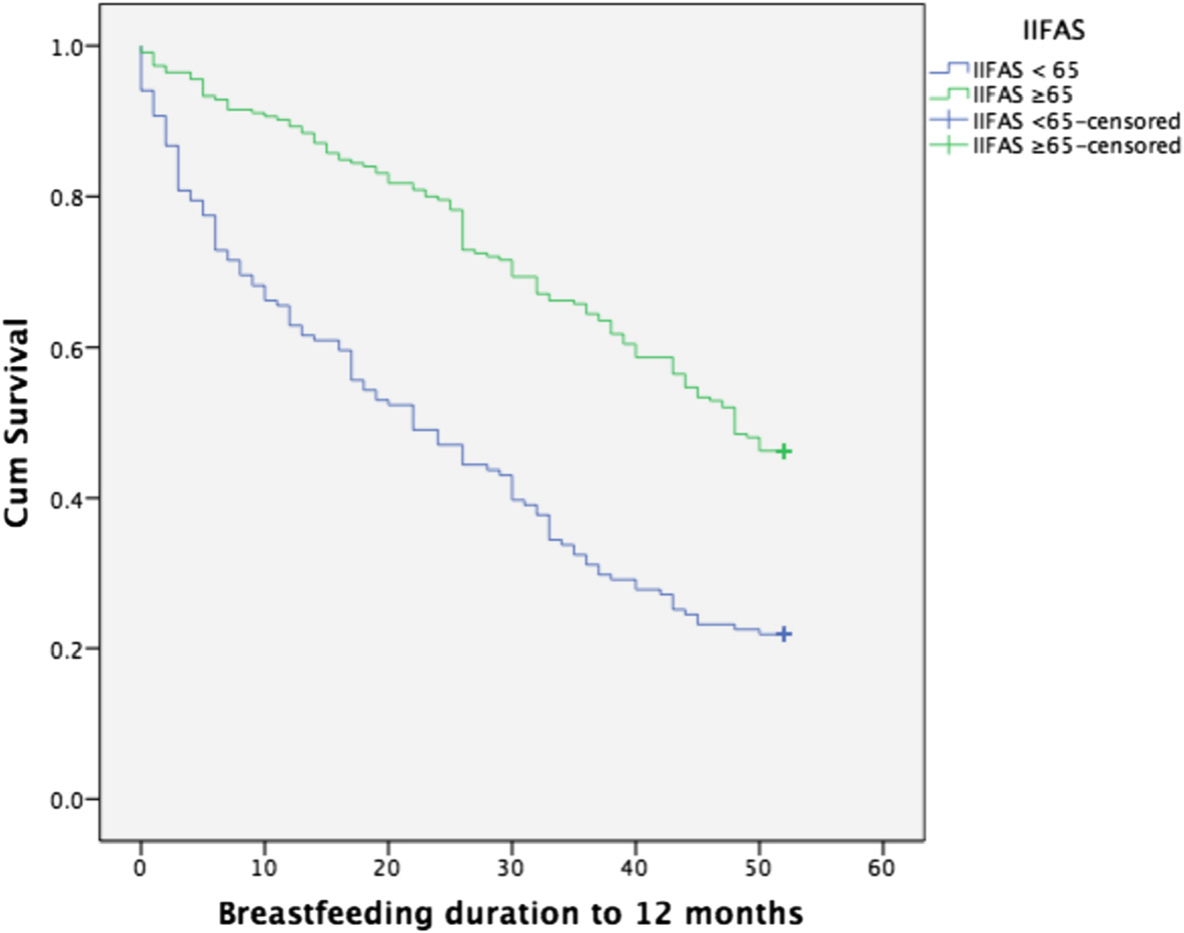
Any breastfeeding duration to 12 months (52 weeks) by IIFAS score

The median duration of exclusive breastfeeding for mothers with an IIFAS score of > 65 was 16 weeks (95 % Confidence Interval (CI) 13.5, 18.5) compared with five weeks for those with a score < 65 (95 % CI 3.2, 6.8) (*p* < 0.0001) (see Fig. 1). The median duration of any breastfeeding to 12 months was more than twice as long for mothers with an IIFAS score > 65 (48 vs. 22 weeks, *p* < 0.001) (see Fig. 2).

After adjusting for potential confounders (see Table 3), mothers infant feeding attitude remained a strong predictor of exclusive breastfeeding to six months, where those with an IIFAS score < 65 were more likely to have ceased (adjusted Hazard Ratio (aHR) 1.81 95 % CI 1.30, 2.51). Similarly, mothers with a IIFAS score < 65 were more likely to have discontinued any breastfeeding at 12 months (aHR 2.38, 95 % CI 1.63, 3.49) (see Table 3).

**Table 3:** Infant feeding attitude variables associated with the risk of discontinuing breastfeeding at 26 and 52 weeks

Variable	EBF (26 weeks)		ABF (26 weeks)		ABF (52 weeks)	
	aHR	95 % CI	aHR	95 % CI	aHR	95 % CI
Mother’s perception of father’s feeding preference						
Ambivalent or prefers bottle-feeding	1.48	1.11, 1.97	1.71	1.02, 2.85	NS	
Prefers breastfeeding (ref)	1.00		1.00			
Mother’s perception of maternal grandmother’s feeding preference						
Ambivalent or prefers bottle-feeding	NS		1.96	1.08, 3.54	2.05	1.37, 3.08
Prefers breastfeeding (ref)			1.00		1.00	
Mother’s Iowa Infant Feeding Attitude Score (IIFAS)						
≤65	1.81	1.30, 2.51	3.45	2.05, 5.81	2.38	1.63, 3.49
> 65 (ref)	1.00		1.00		1.00	

*ABF* Any breastfeeding

*EBF* Exclusive breastfeeding

*NS* Not significant

*aHR* adjusted Hazard Ratio

Variables entered into full model included: maternal age, mother perception of father’s feeding preference, infant’s gender, parity (primiparous/multiparous), infant’s birth weight (< 2500g), whether infant was admitted to SCN, whether mother received conflicting advice in hospital, attendance at antenatal classes, mothers level of education, demand feeding in hospital, fathers occupation, early breast contact, rooming in in hospital, delivery method, grandmothers feeding preference, grandmother’s breastfeeding history, when feeding method was decided, mothers employment in the previous six months, marital status, time to regional centre, mother’s IIAFAS score, age of infant when pacifier introduced, mother’s smoking during pregnancy, planned pregnancy, household income, breastfeeding problems experienced by week four, age of infant when mother returned to work, maternal pre-pregnancy obesity

A total of 54.8 % (*n* = 234) of mothers indicated that their baby’s father preferred breastfeeding, and a further 36.1 % (*n* = 154) indicated that he did not have a preference for either breast or formula feeding. Six women (1.4 %) indicated that their baby’s father preferred formula feeding and 29 women indicated that they had not discussed feeding methods. Mothers who perceived their baby’s father to be ambivalent about breastfeeding or to prefer formula feeding were 48 % more likely to have ceased exclusive breastfeeding and 71 % more likely to have ceased any breastfeeding by 26 weeks, although this was not significant for breastfeeding to 52 weeks (see Table 3).

Approximately one third of mothers (34.9 %, *n* = 149) indicated that their own mother preferred breastfeeding and a further 40.3 % (*n* = 179) indicated that she did not have a preference. Almost one quarter (22.7 %, *n* = 97) reported that they had not discussed infant feeding with their mother and five women (1.2 %) indicated that she preferred formula feeding. Mothers who perceived their own mother to be ambivalent about breastfeeding or prefer formula feeding were 96 % more likely to have ceased any breastfeeding by 26 weeks and more than twice as likely to have ceased any breastfeeding by 52 weeks (see Table 3).

## Discussion

Although studies documenting breastfeeding attitudes have been reported previously in a range of settings [18, 31, 43], this is the first time to our knowledge that the attitudes of women residing in rural areas of Australia have been assessed using the validated IIFAS scale. The finding that women who had a more favourable attitude towards breastfeeding were more likely to exclusively breastfeed to six months concurs with the literature, both in Australia and internationally [13, 15, 43, 44].

Mothers who are predisposed towards breastfeeding are consistently found to have greater duration of breastfeeding regardless of intensity [7, 45]. Those mothers who believe that breastfeeding is more convenient, healthier and cheaper are less likely to introduce formula to their infant than those who find breastfeeding to be embarrassing, restrictive or uncomfortable [9]. In this study, mothers who had positive breastfeeding attitudes were almost twice as likely to be exclusively breastfeeding at six months, compared to mothers with neutral or negative breastfeeding attitudes. This effect was independent of other sociodemographic factors such as age, marital status and education, a finding supported by the literature [11, 15, 18]. Development and implementation of strategies that assist women in developing positive breastfeeding attitudes should be considered as a priority for rural health services, such as providing consistent evidence-based information in a variety of accessible antenatal education settings, to increase skills and confidence in breastfeeding.

The significant independent association between fathers’ infant feeding attitude and breastfeeding duration is also mirrored in findings from other settings [11, 31]. For rural areas, a greater recognition of the role that fathers play in supporting women in the task of breastfeeding is required, and a focus on inclusive antenatal breastfeeding education for fathers should be considered by rural health services. Particular attention should be paid to increasing accessibility of breastfeeding education for parents who are geographically or culturally isolated in rural areas. Similarly, the influence of grandmothers in this cohort on breastfeeding duration is strong and supports the notion that vicarious and practical learning about infant feeding is passed on in this way. Women supporting their daughters to breastfeed in rural areas require consistent evidence-based breastfeeding information in formats that allow them to contribute their own experience and practical assistance.

Whilst it is accepted that positive breastfeeding attitudes are associated with increased breastfeeding duration, there is evidence to suggest that neutrality or ambivalence towards breastfeeding has a negative association [16]. Identifying women who display ambivalence towards breastfeeding antenatally may provide opportunities to influence feeding choice in this period [18], although in this study, more than three quarters (77 %) had decided on their feeding method prior to pregnancy. Further evidence regarding the antenatal breastfeeding attitudes of rural women is required before recommendations about antenatal intervention could be recommended.

The ambivalent attitudes of this cohort to the comparative health benefits of breast milk and formula is of interest, with 40 % expressing a neutral attitude and a further 16 % agreeing that there are no differences in health benefits. It is possible that the proliferation of advertising for “follow-on” or toddler milks has provided a level of reassurance for mothers regarding their choice of infant feeding method. A study examining the influence of advertising of toddler milk products in Australia found that first time mothers perceived these advertisements to be for infant formula and believed the manufacturers claims that formula could provide benefits similar to those normally attributed to breastfeeding based on this information [46]. Whilst Australia has a voluntary system of self-regulation for infant formula manufacturers, based on the World Health Organization’s International Code of Marketing of Breast Milk Substitutes, it appears that the system is failing to protect consumers from advertising that minimises the important differences between breast milk and formula [46]. The rise of social media and networking sites present new challenges in monitoring the pervasiveness of messages promoting infant formula at the expense of the benefits of breastfeeding [47]. To address the apparent perception that formula offers comparative health benefits to breast milk, messages that promote breastfeeding as the normal and optimal way to feed infants are required. Wider community promotion of breastfeeding guidelines, population health benefits and support of breastfeeding goals may help to establish breastfeeding as the normal choice of infant feeding in rural areas and counter these messages to influence the attitudes of women of child-bearing age, as well as their partners and other social supports. Without this general support, breastfeeding messages may be less effective, contributing to the difficulty mothers face in reaching the recommendations for exclusive breastfeeding [48].

### Limitations

The results of this study suggest that the IIFAS is a robust predictor of breastfeeding intention in rural areas of Western Australia, however some caution should be exercised in generalising these findings. Firstly, just over one half of the eligible women contacted participated in the study and significant differences in sociodemographic characteristics between women who completed the study and those who were lost to follow up may have contributed to selection bias in the results. Additionally, few mothers in the sample were from culturally or linguistically diverse backgrounds, however the tool has been used and validated in a number of cultural groups, lending support to its use in non-homogenous settings. A larger sample size to control for loss of under-represented sociodemographic groups is recommended to determine the applicability of these findings.

## Conclusions

Women in rural Western Australia who have a more positive attitude towards breastfeeding are more likely to start and continue to breastfeed. Additionally, positive breastfeeding attitudes of close social supports including the infant’s father and grandmother are significant influences on breastfeeding duration. Whilst this finding mirrors the data reported in metropolitan areas of Australia or indeed in other developed countries, there are unique challenges in ensuring that rural women are provided with consistent, evidence-based breastfeeding information, are presented with positive and supportive community attitudes and are encouraged to begin and continue to breastfeed in order to meet the current WHO recommendations. Further research examining the breastfeeding attitudes of specific subgroups such as men, grandparents and adolescents in rural areas will contribute to the evidence base and help to ensure that breastfeeding is seen as the normal method of infant feeding.

## Abbreviations

IIFAS: Iowa infant feeding attitude scale
WHO: World Health Organization
BFHI: Baby friendly hospital initiative
aHR: adjusted hazard ratio
CI: Confidence interval
